# A novel 72-kDa leukocyte-derived osteoglycin enhances the activation of toll-like receptor 4 and exacerbates cardiac inflammation during viral myocarditis

**DOI:** 10.1007/s00018-016-2423-7

**Published:** 2016-11-23

**Authors:** Marieke Rienks, Anna Papageorgiou, Kristiaan Wouters, Wouter Verhesen, Rick van Leeuwen, Paolo Carai, Georg Summer, Dirk Westermann, Stephane Heymans

**Affiliations:** 1 Center for Heart Failure Research, Cardiovascular Research Institute Maastricht, Universiteitssingel 50, 6229 ER Maastricht, The Netherlands; 2 Molecular and Vascular Biology, Department of Cardiovascular Sciences, KU Leuven, Hamburg, Germany; 3 Centre for Cardiology Research, Hamburg University, Leuven, Belgium

**Keywords:** Glycosylation, TLR4, Osteoglycin, Inflammation, Viral myocarditis

## Abstract

**Background:**

Viral myocarditis can severely damage the myocardium through excessive infiltration of immune cells. Osteoglycin (OGN) is part of the small leucine-rich repeat proteoglycan (SLRP) family. SLRP’s may affect inflammatory and fibrotic processes, but the implication of OGN in cardiac inflammation and the resulting injury upon viral myocarditis is unknown.

**Methods and results:**

This study uncovered a previously unidentified 72-kDa variant of OGN that is predominant in cardiac human and mouse samples of viral myocarditis. Its absence in mice significantly decreased cardiac inflammation and injury in Coxsackievirus-B3-induced myocarditis. It also delayed mortality in lipopolysaccharide-induced endotoxemia going along with a reduced systemic production of pro-inflammatory cytokines. This 72-kDa OGN is expressed in the cell membrane of circulating and resident cardiac macrophages and neutrophils. Co-immunoprecipitation and OGN siRNA experiments revealed that this 72-kDa variant activates the toll-like receptor-4 (TLR4) with a concomitant increase in IL-6, TNF-α, IL-1β, and IL-12 expression. This immune cell activation by OGN occurred via MyD88 and increased phosphorylation of c-jun. Finally, the 72-kDa chondroitin sulfate is the result of O-linked glycosylation of the 32-kDa protein core of OGN. In contrast, the 34-kDa dermatan sulfate-OGN, involved in collagen cross linking, was also the result of O-linked glycosylation.

**Conclusion:**

The current study discovered a novel 72-kDa chondroitin sulfate-OGN that is specific for innate immune cells. This variant is able to bind and activate TLR4. The absence of OGN decreases cytokine production by both circulating and cardiac leukocytes upon (systemic) LPS exposure, and reduces cardiac inflammation and injury in viral myocarditis.

**Electronic supplementary material:**

The online version of this article (doi:10.1007/s00018-016-2423-7) contains supplementary material, which is available to authorized users.

## Introduction

Osteoglycin (OGN) belongs to the small leucine-rich proteoglycan (SLRP) family of proteins, which also includes biglycan and decorin. These SLRPs are well known for their well-timed action on shaping the architecture and organization of collagen-rich extracellular matrices in the heart and other organs. OGN is critical during the wound healing process after myocardial infarction by stimulating the formation of well-aligned collagen fibers in the infarct scar [1]. Some matrix components, proteoglycans, and glycoproteins have been recognized for regulating inflammation in organs outside the heart [2]; however, no such role has been described for OGN in either the heart or other organs. Furthermore, OGN is a proteoglycan and is, therefore, subject to glycosylation, a post-translation modification that adds glycosaminoglycans and glycans to proteins in the endoplasmic reticulum and Golgi apparatus, diversifying protein function [2]. While OGN requires glycosaminoglycan keratan sulfate for corneal transparency [3, 4], no clear-cut and comprehensive description of all potential glycosylation variants is available. Moreover, nothing is known about the glycosylation state of OGN during different forms of cardiac diseases and the impact of this glycosylation on cardiac inflammation or fibrosis.

Adverse cardiac inflammation and damage upon viral infection, followed by adverse remodeling, is an important cause of heart failure and sudden cardiac death in previously healthy individuals [5]. Understanding how the immune system responds to various endangering stimuli (ischemia versus viruses) might clarify the contribution of the inflammatory response to adverse cardiac remodeling and hence lead to the discovery of specific therapeutic strategies. The need for new specific therapeutic strategies is underlined by the poor prognosis once heart failure becomes chronic [6].

Here, we discovered that SLRP OGN can be differentially glycosylated depending on the underlying pathophysiology, inflammation (myocarditis) versus fibrosis (myocardial infarction). Here, we link the OGN-dermatan sulfate (34 kDa) predominantly to cardiac fibrosis, while the previously unknown OGN-chondroitin sulfate (72 kDa) prevails in the cell membrane of both murine and human leukocytes and is thus linked to inflammation. We unveil that this 72-kDa inflammatory variant binds to TLR4 and enhances its activation. As such, increased OGN results in exaggerated innate immune responses in both endotoxemia and viral myocarditis.

## Results

### Different OGN variants are present in different cardiac pathologies

**Fig. 1:**
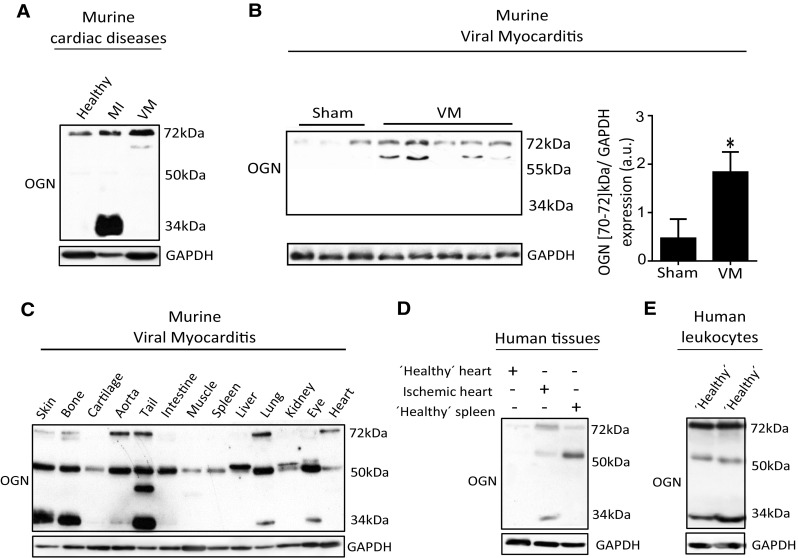
Murine and human tissues express different OGN variants in distinct cardiac diseases. **a** Western blot analysis for OGN revealed a small variant (34 kDa) in murine myocardial infarction, whereas in murine viral myocarditis, a large variant (72 kDa) was present (*n* ≥ 4). **b** Western blot analysis of cardiac tissues of mice 7 days after viral exposure was compared with healthy controls and quantified. Viral infection led to increased expression of a 70–72 kDa OGN variant compared with healthy controls (sham *n* = 3, VM *n* = 5, *p < 0.05). **c** Differential expression of the main OGN variants in various healthy murine tissues. **d** Western blot analysis for OGN in human ischemic myocardial tissue showing increased expression of the large (72 kDa) and the small (34 kDa) OGN variants relative to those in the control cardiac tissue. **e** Human buffy coat revealed a similar presence of the OGN variants with high abundance of the 72-kDa variant, which was barely detectable in human splenic tissues

**Fig. 2:**
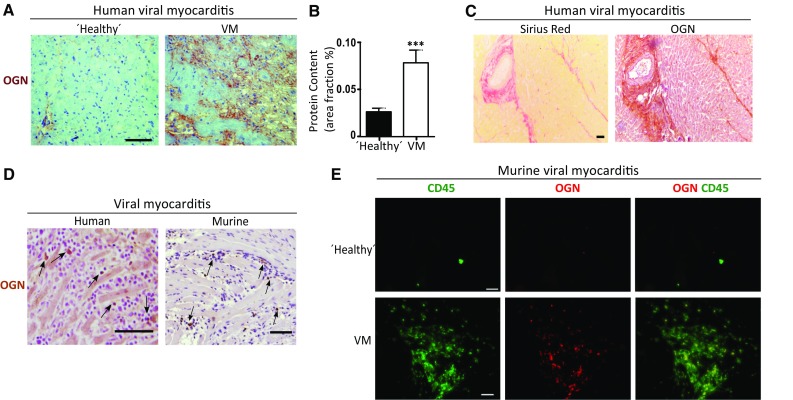
Different OGN variants are correlated with fibrosis and inflammation in murine and human cardiac diseases. **a** Increased immunohistochemical OGN staining was found in patients diagnosed with myocarditis. **b** Quantification of OGN staining in patients diagnosed with myocarditis (*n* ≥ 14). **c** OGN expression in human myocarditis biopsies coincided with fibrosis as shown by Sirius red staining. **d** OGN staining was also found on leukocytes in human myocarditis biopsies as well as in the hearts of mice subjected to CVB3-induced myocarditis. **e** Immunofluorescence demonstrated the co-localization of OGN with CD45+ leukocytes in the hearts of mice subjected to virus myocarditis compared with healthy controls (*scale bar* healthy controls: 20 µm). All experiments were repeated at least twice. *Scale bar* 50 µm. *VM* viral myocarditis, *MI* myocardial infarction

First, protein expression of OGN was studied in murine hearts 7 days after intraperitoneal injection of Coxsackie B3 virus (CVB3). The sole presence of a new yet undescribed cardiac OGN variant of approximately 72-kDa was found in the murine hearts with viral myocarditis (Fig. 1a). Contrarily, a 34-kDa OGN variant was highly abundant in murine myocardial infarcts (Fig. 1a), temporally and spatially associated with collagen fibrillogenesis [7, 8]. Expression of this 72-kDa protein variant increased significantly in the hearts of WT mice after inoculation with CVB3 compared with healthy controls (Fig. 1b). When further analyzing OGN protein expression in different tissues, we identified very distinct yet consistent glycosylation patterns. Murine corneal tissues had a high abundance of the 50-kDa keratan sulfate variant [3] which is in line with literature findings. Tissues with high collagen and connective tissue contents, such as bone, tail, and skin, presented with the 34-kDa variant (Fig. 1c). Furthermore, human tissue and blood samples demonstrated a comparable occurrence of the OGN protein variants. In post-mortem left ventricular tissues from ischemic patients in which the collagen content, as a consequence of scaring and fibrosis, is high, mainly the 34-kDa OGN variant was detected, while it could not be found in control patients without scaring (Fig. 1d). This is supported by immunohistochemical staining for OGN in human myocardial infarcts, revealing that OGN staining clearly matched cardiac fibrosis [9]. In sharp contrast, the 72-kDa variant was highly expressed on human circulating leukocytes (Fig. 1e), whereas it could not be detected in post-mortem splenic tissue (Fig. 1d). Therefore, we hypothesized that the presence of distinct OGN variants may be associated with the specific underlying pathophysiological processes, reactive to an environmental pathogen. In viral myocarditis patients, where there is both fibrosis and inflammation [10], we identified an increase in OGN staining compared with control hearts (Fig. 2a, b). Here, positive OGN staining was found in areas with fibrosis (Fig. 2c) but also on immune cells (Fig. 2d). Correspondingly, in our murine model of CVB3 myocarditis, where inflammation is present, but fibrosis is not [11], OGN staining predominated on leukocytes (Fig. 2e). Consequently, the observed variation in size of this proteoglycan in relation to either cardiac fibrosis (34-kDa) or cardiac inflammation (72-kDa) led us to conclude that differential glycosylation may be in control.

### OGN is present on cardiac and circulating innate immune cells

**Fig. 3:**
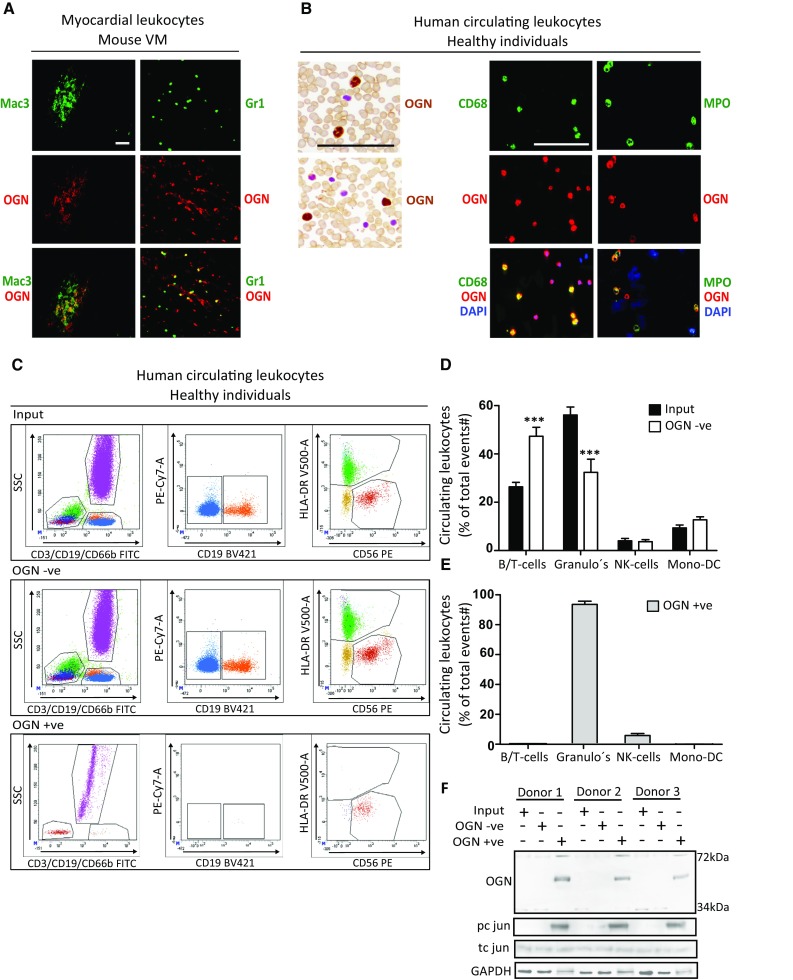
72-kDa iOGN was found on cardiac and circulating innate immune cells. **a** Murine cardiac monocytes/macrophages (Mac-3) and neutrophils (Gr1) expressed OGN during CVB3-induced myocarditis. **b** Circulating human monocytes and neutrophils also expressed OGN. **c** Representative FACS plots of total human circulating leukocytes, OGN-negative (OGN −ve), and -positive (OGN +ve) circulating leukocytes. **d**, **e** FACS analysis revealed that the OGN-positive circulating leukocytes consisted mostly of neutrophils. **f** OGN-positive circulating immune cells had increased phosphorylation of c jun. *n* = 4, *p < 0.001; all experiments were repeated at least twice. *Scale bar* 50 µm

As the 72-kDa ‘inflammatory’ OGN (iOGN) was primarily found in viral myocarditis on leukocytes, we further analyzed its specific expression in circulatory leukocytes versus cardiac leukocytes using immunofluorescence and flow cytometry in both mice and humans. OGN clearly co-stained with both murine neutrophil (Gr1)- and monocyte/macrophage (Mac3)-markers in the myocardium (Fig. 3a). To verify that the relevance of OGN was not exclusive for mice, we confirmed its occurrence on circulating human leukocytes, the source for cardiac inflammation in response to CVB3 infection. Human peripheral leukocytes in blood smears and buffy coats demonstrated a similar co-localization of OGN with human peripheral neutrophil- and monocyte-markers (Fig. 3b). Separating OGN-positive peripheral leukocytes (OGN +ve) from the total circulating peripheral leukocytes using OGN-bound magnetic Dynabeads allowed us to better identify the OGN expressing circulating immune cell population by FACS analysis. As approximately 50–70% of all human circulating leukocytes are composed of neutrophils, FACS analyses revealed the presence of OGN predominantly on peripheral neutrophils (Fig. 3c–e). Interestingly, these circulating iOGN-positive leukocytes showed significantly more phosphorylation of c-jun than the iOGN negative leukocytes, suggesting very distinct phenotypic characteristics for these iOGN-positive innate immune cells (Fig. 3f).

### Glycosylation leads to different OGN variants

**Fig. 4:**
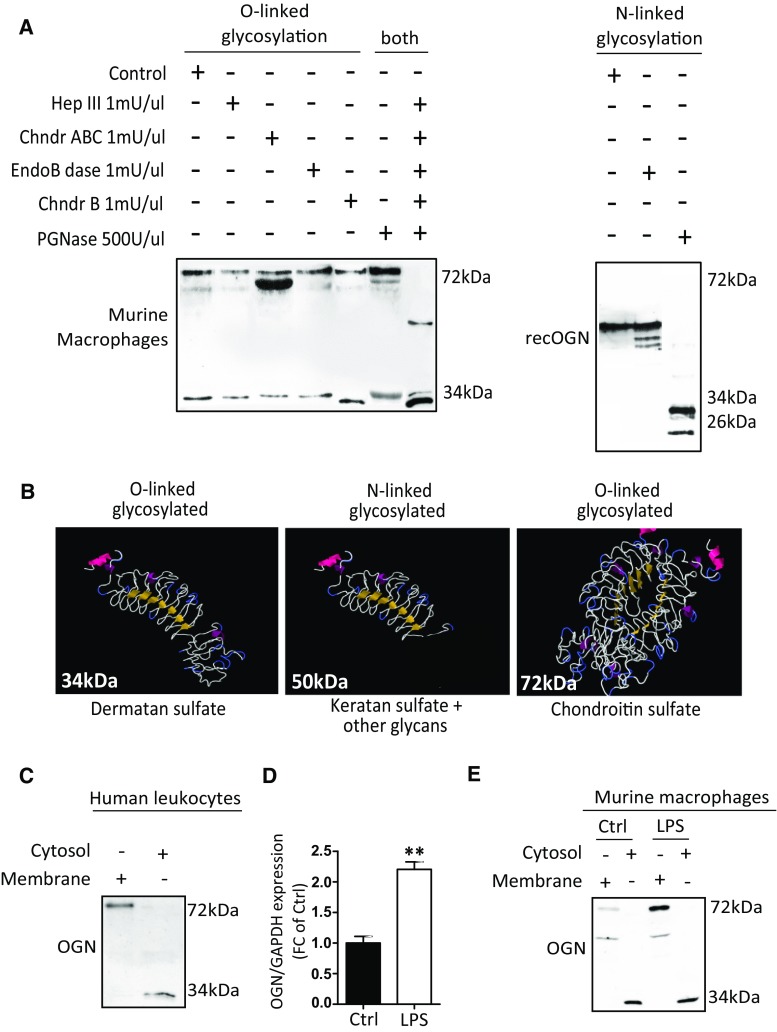
Glycosylation of the 32-kDa OGN core protein results in the production of different protein variants. **a** Enzyme treatment of macrophage protein lysates revealed the differential presence of glycans and glycosaminoglycans; treatment with chondroitinase ABC reduced the size of the 72-kDa protein variant, indicating that chondroitin and dermatan sulfate is attached, whereas treatment with chondroitinase B only slightly reduced the size of the 34-kDa variant, indicating that it only has dermatan sulfate attached. The simultaneous addition of all of the enzymes reduced the protein glycosylation of both the 34-kDa and 72-kDa variants entirely. Treatment of the 50-kDa OGN variant with PGNase reduced the size detected on western blot significantly, indicating that it is N-linked glycosylated. **b** Predicted structures of the OGN variants and their respective glycosylations. **c** Cell fractionation of fresh human buffy coat lysates revealed the presence of the 72-kDa OGN variant in the cell membrane, whereas the 34-kDa variant was found in the cytosol. **d**, **e** In mouse bone marrow-derived macrophages, LPS stimulation increased the expression of OGN in the cell membrane. All experiments were repeated at least twice

Because glycosylation is a very dynamic step that adds glycans or glycosaminoglycans to proteins, we explored whether the production of these different OGNs in the heart (34, 50 and 72 kDa) originated from the single 32-kDa protein backbone. Therefore, we treated protein lysates with specific enzymes that are able to cleave the different glycosaminoglycans or glycans. We found that the 50-kDa OGN has N-linked glycans and N-linked keratan sulfate attached (Fig. 4a). The addition of O-linked dermatan sulfate resulted in the formation of the 34-kDa variant (Fig. 4a). Furthermore, the addition of chondroitin sulfate led to the formation of the 72-kDa variant, which is potentially a dimer that could only be reduced by treatment with all enzymes simultaneously (Fig. 4a). In brief, the 32-kDa protein core of OGN can be post-translationally modified by either O- or N-linked glycosylation (Fig. 4b).

Interestingly, whereas the 72-kDa inflammatory OGN (iOGN) variant was primarily located in the cell membrane of both human peripheral leukocytes and murine bone marrow-derived macrophages (BMDMs), the 34-kDa fibrosis OGN (fOGN) was located in the cytosol, as revealed by cell fractionation (Fig. 4c, e). Furthermore, stimulation of BMDMs with lipopolysaccharides (LPS) not only resulted in a significant increase in the transcript levels of OGN (Fig. 4d), protein expression of the 72-kDa iOGN membrane variant also increased in response to LPS (Fig. 4e). Furthermore, the transcription and translation of OGN were paralleled by an increased gene expression of key glycosylation enzymes, such as Xylosyl transferase 2 (XYLT2) and d-glucuronyl C5*-*epimerase (Supplementary Fig. 1). LPS thus increases the transcription, translation, and glycosylation of OGN.

### Membrane-bound inflammatory iOGN interacts with TLR4 in leukocytes

**Fig. 5:**
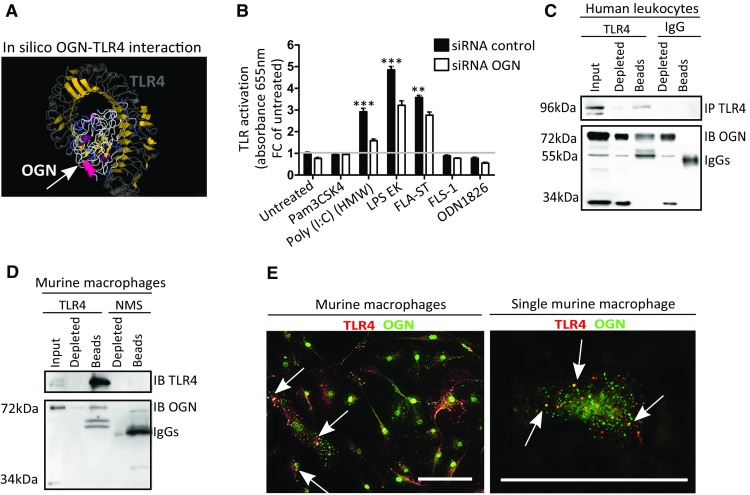
Membrane bound iOGN interacts with TLR4 on leukocytes. **a** Docking predictions of TLR4 and OGN reveal a potential interaction between these proteins via their leucine-rich repeats. **b** HEK-Blue™-mTLR cells containing an inducible SEAP reporter gene were stimulated with TLR-specific ligands (Pam3CSK4 for TLR1/2, Poly(I:C) (HMW) for TLR3, LPS-EK for TLR4, FLA-ST for TLR5, FLS-1 for TLR6/2, and ODN1826 for TLR9) with and without OGN knockdown. OGN knockdown reduced the activation of TLR3, -4 and -5. **c**, **d** OGN co-immunoprecipitated with TLR4 in human peripheral leukocytes and murine bone marrow-derived macrophages. **e** Confocal immunofluorescence revealing the co-localization of OGN with TLR4 in primary murine macrophages. *n* ≥ 4, *p < 0.001. All experiments were repeated at least twice. *Scale bar* 50 µm

Next, we sought to accurately understand the biological role of the 72-kDa iOGN that was present on innate immune cells where it was responsible for the apparent “active” phenotypic appearance of these cells. Given the ability of leucine-rich repeats to interact with TLRs [12], we speculated that this 72-kDa membrane-anchored OGN might influence TLR signaling. In silico structure modeling predicted a potential interaction between the concave leucine-rich surface of OGN and the extracellular LRR domain of TLR4 that has been implicated in CVB3-myocarditis disease severity [13, 14] (Fig. 5a). Therefore, HEK-Blue™-mTLR cells containing an inducible secreted embryonic alkaline phosphatase (SEAP) reporter gene were stimulated with TLR-specific ligands (Pam3CSK4 for TLR1/2, Poly(I:C) (HMW) for TLR3, LPS-EK for TLR4, FLA-ST for TLR5, FLS-1 for TLR6/2, and ODN1826 for TLR9) with and without OGN knockdown (Supplementary Fig. 2A). OGN knockdown significantly decreased TLR3, -4 and -5 signaling (Fig. 5b), supporting a possible interaction between OGN and TLRs. Where several studies describe the role of TLR4 during viral infection, as TLR4 is upregulated in response to CVB3 inoculation on the plasma membrane [5, 15] and contributes to its pathogenesis [16], the contribution of especially TLR3 and TLR5 is less founded [17]. Therefore, we focused on investigating the interaction of OGN and TLR4 and found that OGN co-immunoprecipitated with TLR4 in both human peripheral leukocytes as well as in BMDM lysates (Fig. 5c, d). In line with this observation, immunofluorescence showed co-expression of OGN with TLR4 on primary macrophages (Fig. 5e). Nevertheless, it would be interesting to explore in a future study whether there is overlap or specificity in OGN-TLR activity.

As approximately 8 ± 2% of the human peripheral circulating leukocytes expressing OGN on their surface appear to be more “activated” (Fig. 4d–f), we wondered whether this may be a consequence of augmented TLR4 signaling. To validate the interaction of OGN with TLR4 in vivo, we reasoned that a lack of OGN on circulating leukocytes in OGN null mice, may alter TLR4 signaling in the mouse model of endotoxemia. We, therefore, subjected OGN WT and KO mice to endotoxemia, a model of septic shock where TLR4 activation is, in part, driving pathogenesis. Although mortality was severe in both genotypes, OGN WT mice died earlier than their OGN-KO littermates (Table 1, *p* = 0.05; Median survival: 8 h in WT versus 14 h in KO; ratio 0.5714 with 95% CI of 0.030–1.113). The serum levels of IL-12 and IL-1β were also measured and significantly higher in OGN WT mice than in their KO littermates 1 h after receiving an intravenous dose of LPS (Table 1). Because only a small subset of circulating leukocytes expressed iOGN, no clear differences were found in the serum TNFα and IL-6 levels (Table 1). Collectively, these findings suggest that iOGN present on innate immune cells is crucial for boosting TLR4 activation.

**Table 1:** OGN boosts TLR4 activation in endotoxemia, a model of septic shock

	WT (*n* = 23)	KO (*n* = 22)	
Median survival (h)	8	14	Ratio 0,57 (95% CI of ratio 0.03–1.11) Log-rank (Mantel Cox) test *p* = 0.05
TNFα (pg/ml)	5796 ± 570.5	5862 ± 505.1	*p* = 0.90
IL-6 (pg/ml)	56,191 ± 237.9	55,820 ± 294.4	*p* = 0.30
IL-12 (pg/ml)	396.3 ± 25.7	320 ± 24.4	*p* = 0.03
IL-1β (pg/ml)	17.5 ± 1.5	12,5 ± 1.4	*p* = 0.02

### OGN promotes TLR4 activation resulting in enhanced MAPK-induced cytokine production

**Fig. 6:**
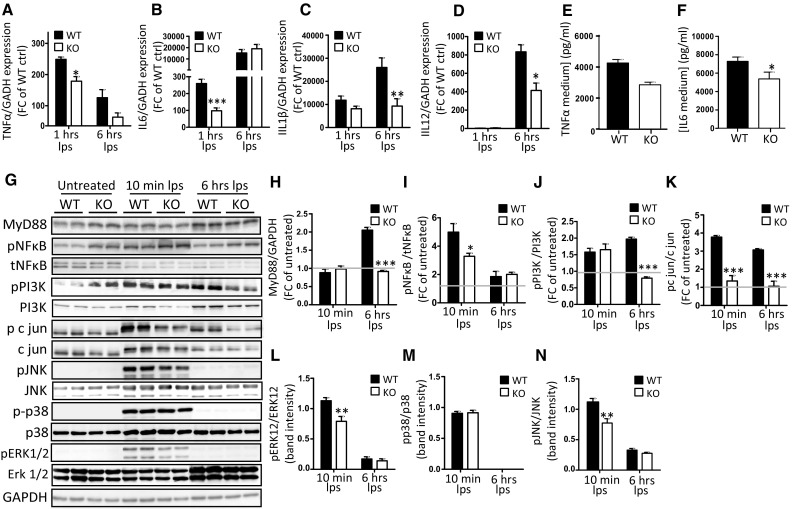
OGN promotes TLR4 activation by enhancing MAPK-induced cytokine production. Bone marrow-derived macrophages (BMDMs) from WT and KO mice were isolated and stimulated with LPS (10 ng/ml). **a**–**d** LPS stimulation resulted in the blunted induction of pro-inflammatory cytokines TNFα and IL-6 expression (1 h) as well as IL-1β and IL-12 at a later stage (6 h) (**c**, **d**). This difference in TLR4 activation was reflected in the lower TNFα and IL-6 expression levels in the medium of OGN-KO BMDMs (**e**, **f**). This increased cytokine production was the result of significant induction of MyD88 that was blunted in the OGN-KO BMDMs (**g**, **h**). **g** Western blot analysis and further quantification revealed blunted c-jun, ERK1/2 and JNK phosphorylation in the KO BMDMs, and blunted pNFkB induction (**i**–**l**). All experiments were repeated at least twice. *n* ≥ 3; *p < 0.05; **p < 0.01; ***p < 0.001

As TLR4 activation leads to the expression of pro-inflammatory cytokines due to phosphorylation of many downstream kinases, we wanted to confirm that the TLR4-OGN interaction influenced TLR4 signaling. By stimulating isolated WT and KO macrophages with LPS, we first analyzed TLR4 activation by determining pro-inflammatory cytokine expression. The transcript levels of IL-6 and TNFα were significantly increased in WT macrophages 1 h after LPS stimulation (Fig. 6a, b), resulting in increased protein levels of these cytokines in the medium 6 h after stimulation (*p* < 0.05, Fig. 6e, g). Concordantly, RNA expression of IL-1β and IL-12 in OGN WT macrophages was significantly higher 6 h after LPS stimulation (Fig. 6c, d). Next, we examined whether OGN could influence the phosphorylation of several kinases that are needed for TLR4 activation. KO macrophages lacked the significant MyD88 induction that was apparent in WT macrophages in response to LPS (*p* < 0.001, Fig. 6f, h, Supplementary Fig. 3 which includes 1 h after LPS stimulation). While the baseline expression of pNFkB was greater in OGN-KO macrophages, the induction was far less that in the WT macrophages. Furthermore, a striking increase of c-jun phosphorylation was found in response to LPS in WT compared with KO macrophages, as a consequence of increased MAPK phosphorylation (*p* < 0.001, Fig. 6f, i–l). Thus, our findings confirm that the interaction of OGN with TLR4 increases phosphorylation c-jun which results in more TLR4 activation and hence pro-inflammatory cytokine expression (Fig. 8).

### iOGN present on innate immune cells during viral myocarditis aggravates cardiac inflammation

**Fig. 7:**
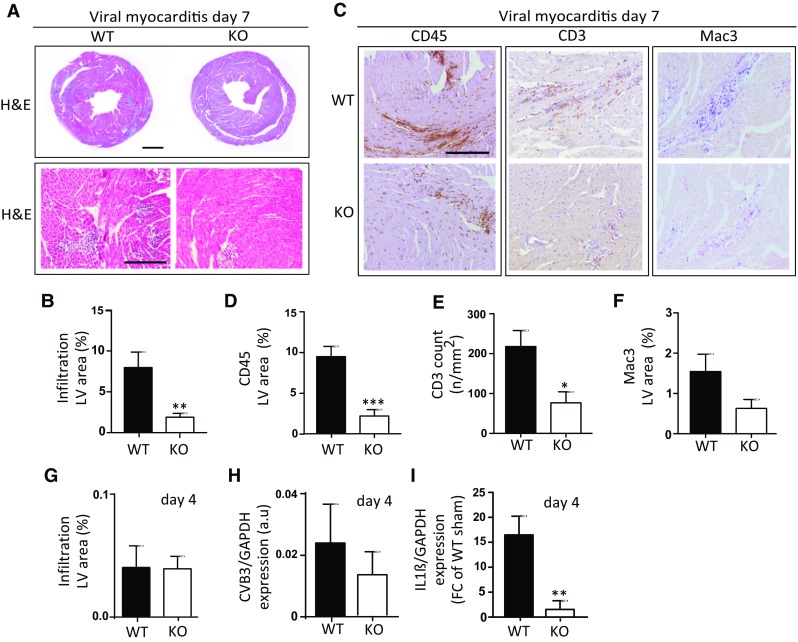
OGN increases cardiac inflammation in CVB3-induced murine myocarditis. Analysis of H&E staining in OGN-KO mice revealed significantly reduced cellular infiltration relative to WT mice in human CVB3-induced viral myocarditis (**a**, scale: 1 mm *top* and 50 µm *bottom*) and **b** quantification (*n* ≥ 11, **p < 0.01). **c** Decreased immune infiltration in OGN-KO mice was confirmed by the reduced presence of CD45 positive leukocytes (**d**), CD3 positive lymphocytes (**e**), and Mac3 positive macrophages (**f**). **g** Four days after viral inoculation, the amount of immune cell infiltration was still low and comparable in both genotypes (*n* ≥ 4). **h** Viral levels analyzed by RT-PCR were also comparable at this time. **i** Pro-inflammatory cytokine IL-1β RNA expression, on the other hand, was already significantly increased in WT compared with KO mice 4 days after viral exposure. *Scale* 50 µm

Next, we wondered whether the lack of this OGN–TLR4 interaction may influence cardiac inflammation as lack of TLR4 signaling during viral myocarditis has previously been associated with decreased cardiac inflammation [16, 18]. Therefore, we subjected male OGN WT and KO mice to the murine model of CVB3-induced myocarditis. The absence of OGN in KO mice significantly reduced cardiac immune cell infiltration relative to that of WT mice 7 days after viral exposure (8 ± 1.89% in OGN WT, *n* = 11 vs 1.9 ± 0.4% in KO, *n* = 9; *p* = 0.01) (Fig. 7a, b). More specifically, infiltration of leukocytes (CD45), lymphocytes (CD3) and macrophages (Mac3) decreased in the absence of OGN (Fig. 7c–f), supporting the pro-inflammatory role of OGN in myocarditis through enhanced TLR4 signaling. Furthermore, while CVB3 viral levels and immune cell recruitment were still similar in the heart at day 4, IL-1β expression was already significantly increased in the hearts of OGN WT animals, again supporting enhanced TLR4 activation in the presence of iOGN (Fig. 7g–i). In conclusion, the absence of OGN decreases immune cell infiltration in CVB3-infected murine hearts, suggesting that increased expression of leukocyte-specific 72 kDa OGN in myocarditis aggravates cardiac inflammation in the acute phase of myocarditis.

## Discussion

This study has identified three major OGN variants that are formed via either N- or O-linked glycosylation and correlated with distinct pathophysiological processes. First, the attachment of N-linked glycans and keratan sulfate to the 50-kDa OGN variant predominates in the eye, where it is important for corneal transparency [3]. The smallest 34-kDa dermatan sulfate-OGN variant is highly abundant in fibrotic tissues, as in cardiac infarct scars [9]. This fibrotic dermatan sulfate-OGN (fOGN, 34 kDa) is needed for proper collagen fibrillogenesis in the heart [7, 8], which is in line with previous observations where dermatan sulfate itself is involved in kidney fibrosis [19, 20]. Interestingly, we uncovered a new O-linked chondroitin sulfate-OGN variant (iOGN, 72-kDa) that is present in the membrane of immune cells, where it binds and activates TLRs. Chondroitin sulfate binding to the protein backbone of OGN may result in putative dimerization as predicted in silico by us and supported by previous studies for other SLRPs [21, 22]. Collectively, these data indicate that a matrix element can have distinct appearances and roles in different disease contexts due to distinctive glycosylation, significantly increasing our understanding of the versatile nature of the ECM. Furthermore, as glycosylation is important for the sorting and distribution of proteins within the cell [23, 24], it may also explain the transportation of OGN to either the cell membrane (iOGN) or extracellular environment (fOGN). However, it is still unclear what drives these protein glycosylation processes, as the serial and mutual role of differentially expressed glycosylation enzymes has not been completely elucidated. Hence, at present, proper tools to study these spatio-temporal differences in protein glycosylation are limited.

**Fig. 8:**
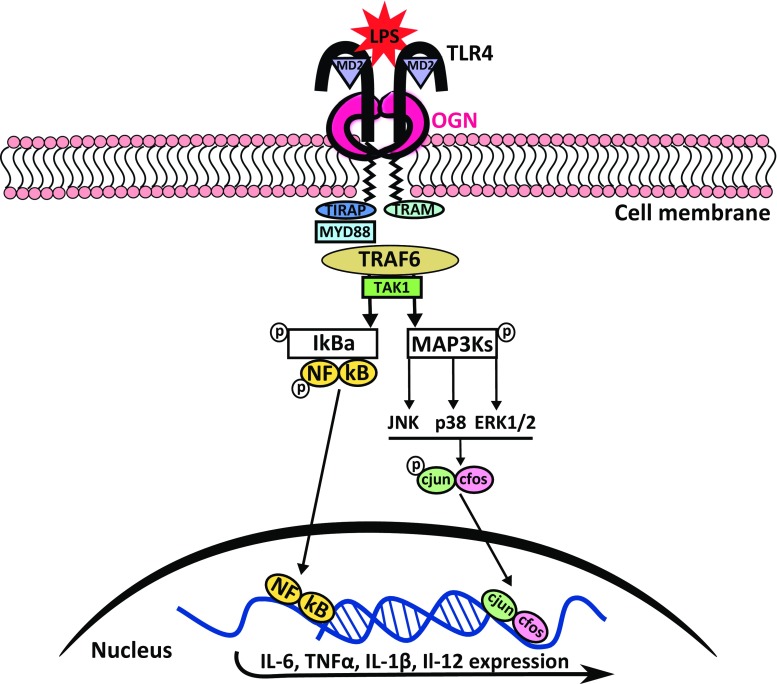
Schematic overview of the proposed iOGN:TLR4 signaling pathway. TLR4 stimulation with LPS results in receptor dimerization and subsequent high-affinity binding of the bridging adaptor molecules TRAM and TIRAP. The bound and activated MyD88 then activates IRAK4, TRAF6, TAK1, and IKK complexes, and TRIF signals through RIP1 to TRAF6/TAK1 and IKK. Both of these pathways result in increased pro-inflammatory cytokine expression. The phosphorylation of MAPKs by TAK1 is enhanced by OGN, resulting in increased c-jun phosphorylation with subsequent nuclear translocation of AP1 (the phosphorylated c-jun/c fos complex), which again resulted in increased pro-inflammatory cytokine expression, reinforcing the inflammatory response [47]

Nonetheless, we found that this 72-kDa iOGN is present on innate immune cells in the heart and in the circulation, where it can bind and activate TLR4 (Fig. 8). This activation results in an aggravated immune response in CVB3 induced viral myocarditis. Interestingly, there is no clear co-localization of OGN with CD45 positive cells residing in the healthy myocardium suggesting that they migrate from the circulation into the heart upon injury. Even more, the increased expression of IL-1β in the WT mice 4 days after viral inoculation further supports the potential exaggerated immune response we see in OGN WT mice as a consequence of enhanced TLR4 signaling. The clear decrease in IL-1β and IL-18 levels in the heart of TLR4 deficient mice infected with CVB3 indicates that TLR4 signaling increases inflammation in the heart especially via IL-1β induction [25, 26], and hence our interest in this specific cytokine. This may, in part, explain the decreased cardiac inflammation and necrosis in myocarditis in the absence of OGN.

Our findings are in line with the previous studies, revealing that distinct SLRPs, such as decorin and biglycan, directly stimulate TLRs [27, 28]. Moreover, it has been speculated that SLRPs like biglycan may cluster TLRs and influence their downstream signaling events [29], though, where biglycan acts as direct endogenous ligand of TLR4, OGN acts more like a co-receptor influencing TLR4 signaling in the cell membrane. Aside from influencing cardiac inflammation in viral myocarditis, the interaction of OGN with TLR4 may also influence the development and progression of other cardiac diseases. Therefore, as TLR signaling contributes to the pathogenesis of ischemic myocardial injury [30–32], myocarditis [31–33], septic cardiomyopathy [31–33], atherosclerosis [31, 34] and, most importantly, the progression of heart failure [31, 32], therapeutically modifying these receptors seems promising. In patients, however, the results have been mixed. While, monoclonal antibodies against TLR2 (OPN-305) have been beneficial in reducing ischemia–reperfusion injury in pigs [35] and are safe and well tolerated in healthy volunteers [36], they still need to be tested in Phase II clinical trials. In comparison, despite promising preclinical studies [37, 38], no clear benefit was identified for the TLR4-specific antagonist Eritoran in elective cardiac surgery patients in a Phase II, double-blind, placebo-controlled study [39]. The failure of such studies emphasizes the complexity of TLR activation and highlights the need to better understand the exact mechanisms utilized by host-derived ligands [40]. Therefore, unraveling the complete collection of exogenous and endogenous ligands in the extracellular space that interact with TLRs is vital and will help in comprehending their contribution to cardiac inflammation in cardiac disease. Furthermore, since TLR3 is clearly implicated in immune activation in viral myocarditis [41], exploration of OGN and TLR3 and 5 interactions is needed to unravel the precise effect of OGN on cardiac inflammation in different cardiac diseases.

Finally, we found that in humans, iOGN was expressed on migrated myocardial as well as circulating leukocytes, where expression was found on macrophages and on a subset of circulating neutrophils displaying a more pro-inflammatory phenotype. This observed phenotype with accompanying functional characteristics, therefore, may contribute to the development of inflammatory cardiac diseases, which has already been recognized for specific neutrophil subsets during disease [15, 42].

In conclusion, we have identified for the first time that SLRP OGN can be glycosylated in multiple ways, resulting in the production a dermatan sulfate glycosylated variant (fOGN) that is associated with fibrosis and a chondroitin sulfate glycosylated membrane-bound variant (iOGN) that is linked to inflammation. This iOGN is present in circulating and cardiac innate immune cells where it boosts TLR4 signaling, as in viral myocarditis and septic shock.

## Materials and methods

### Animals

C57Bl6/J male and female *OGN*-KO mice (backcrossed more than 15 times) and WT mice in the same background between 8 and 12 weeks of age were used in this study, and all experiments were performed using age and sex-matched groups. Mice were maintained in specific pathogen-free facilities at Leuven University. All of the study protocols were approved by the Animal Care and Use Committee of the University of Leuven 243/2013. Experiments were performed according to the official rules formulated in the Belgian law on the care and use of experimental animals.

### Murine CVB3-induced viral myocarditis model

Eight- to twelve-week old *OGN*-KO and WT mice were inoculated by intraperitoneal (ip) injection of 1 × 10^7^ cells with a 50% infective dose (CCID_50_) of CVB3 (Nancy Strain) diluted in 0.2 mL of saline on day 0. All animals were anaesthetized with an ip injection of Xylazine (10 mg/kg) and Ketamine (100 mg/kg) and sacrificed by cervical dislocation 2, 4 or 7 days after CVB3 infection. Organs were excised for further molecular and histological analyses.

### Endotoxic shock model and cytokine measurements

Age- and sex-matched OGN WT and KO mice between 6 and 12 weeks of age were given an ip injection of 40 mg of D-galactosamine (200 µl) followed by an intravenous injection of LPS (*E. coli* Ultrapure O111:B4, InvivoGen) after 15 min. One hour later, whole blood was taken via eye puncture, after which survival was monitored every hour. After allowing the blood to clot by leaving it undisturbed at room temperature for approximately 30 min, the clot was removed by centrifuging at 1000–2000*g* for 10 min in a refrigerated centrifuge. Serum was stored at −20 °C prior to cytokine measurements. Cytokines were measured in serum and medium from in vitro macrophage experiments using V-PLEX Pro-inflammatory Panel1 (mouse) Kit (K15048D-1) according to the manufacturer’s protocol.

### Staining and immunohistochemistry

Hearts were perfused from the apex with PBS, fixed overnight in zinc fixative (BD Pharmingen, 550523) and processed the following day prior to being embedded in paraffin. The paraffin embedded left ventricle was cut longitudinally in 4-μm sections and stained with hematoxylin and eosin (necrosis/inflammation) or Sirius red F3BA (fibrosis).

Immunohistochemistry on zinc-fixed paraffin sections was performed without using antigen retrieval according to a protocol using antibodies against CD45 (BD Pharmingen; 1:200), Mac3 (Serotec MCAP497; 1:100), CD3 (Thermo Scientific; 1:200), TLR4 (Abcam, ab22048), and OGN (for human slides, Sigma HPA013132, 1:200; for murine slides, US Biological O8062-05A, 1:100). Images were acquired using the Leica Qwin image processing software (Leica, Germany).

### Western blotting and enzyme treatments

Tissues were lysed in RIPA SDS (50 mM Tris–HCl, 150 mM NaCl, 0.1% SDS, 0.5% sodium deoxycholate, 1% NP40, Proteinase Inhibitor Cocktail, Roche, 11697498001 and 0.5 mM orthovanadate), after which the protein concentration was determined using a Micro BCA Protein assay kit (Thermo Scientific, Lot MJ162220). Samples were diluted to a final concentration of 2 µg/ul, after which two times sample buffer (25 ml 0.5 M Tris–HCl, 20 ml 100% glycerol, 20 ml, 20% SDS, 35 ml Aqua Dest with 1:10 β-Mercaptoethanol) was added to protein samples at a 1:1 ratio. Cells were directly lysed in sample buffer. For western blot analyses, 20 µg of protein was loaded on a 10% gel (4 ml Aqua Dest, 3.3 ml 30% bisacrylamide, 2.5 ml 1.5 M Tris–HCl, pH 8.8, 0.1 ml 10% SDS, 0.004 ml TEMED). SDS PAGE was performed at 150 V for approximately 90 min, after which the gel was transferred to a PVDF membrane by blotting at 200 mA for 2 h. The membranes were blocked with 3.5% protifar (Nutricia) for 1 h. Primary antibody was incubated overnight in 5% BSA for osteoglycin (R&D, MAB2949), MyD88 (CTS, #4283S) (p)NFkB (CTS #8242 and #3033), (p)PI3 K (CTS, #4292, and #4228), (p)ERK1/2 (CTS, #9101S, and #4695P), (p)JNK (CTS, #9251, and #9252), (p)p38 (CTS, #4631S, and #9212), and (p)c-jun (CTS, #3270P, and #9165P). Secondary antibodies conjugated with horseradish peroxidase (HRP) against rabbit (CTS, #7074S), mouse (CTS, #7076S), or rat (Santa Cruz, E2313) were next detected using enhanced chemi-luminescence, visualized with an Artemis CCD Camera, and quantified using ImageJ.

Enzyme treatments were carried out on macrophage cell lysates that were lysed in RIPA SDS. After determining the protein concentration of the sample with a Micro BCA Protein assay kit, 10 µg of protein lysate (10 μl) was denaturated by the addition of denaturation buffer (supplied with PGNase; Bioke, P0705S) and heated for 3 min at 100 °C. Then, enzymes were added to the lysate and incubated according to the standard protocol (PGNase, Biolabs P0705S or Current Protocol in Molecular Biology, 17.13B ^8^). The enzymes used were Chondroitinase ABC (Sigma, C3667), Endo-B-galactidase (Sigma, C6920), Heparinase III (Sigma, H8891), Chondroitinase B (Sigma, C8058), and PGNase (Bioke, P0705S). For every enzyme, a control sample was incubated at the same temperature and time. Finally, protein glycosylation was analyzed by immunoblotting to determine size changes.

### Structure and docking prediction

We used the I-TASSER Web service (MM1 [43], MM2 [44], and MM3 [45]) to predict the 3D structure of OGN (on 2014-07-12). The default parameters for I-TASSER were used. As an input sequence for OGN, the amino-acid sequence from UniProt (Q62000) was used. The docking prediction of TLR4 (PDB ID: 2Z64, chain A) and OGN was calculated using the Hex Web service (MM4 [46]). Again, the default parameters were used. Visualization of the resulting structures was performed in Jmol (14.2.2, http://www.jmol.org/).

### Co-immunoprecipitation and cell fractionation

Fifty microliters of sheep anti-mouse M280 Dynabeads (Invitrogen, 11201D) was prepared according to the manufacturer’s protocol and incubated with either 4 µg of anti-TLR4 (Abcam, ab22048) or 4 µg of normal mouse serum (NMS; Sigma, M5905) in 50 µl for 2 h at 4 °C. One bacterial plate with macrophages was lysed in PBS, spun down for 10 min at 1200 rpm, and dissolved in 5 ml of IP buffer (150 mM NaCl, 20 mM Tris–HCl, 5 mM EDTA, 1% Triton + Proteinase Inhibitor Cocktail, Roche, 11697498001) for 1 h at 4 °C in a head-to-head rotator. After determining the protein concentration (Thermo Scientific, Lot MJ162220), 300 µg of protein lysate in 200 µl of IP buffer was mixed with preincubated TLR4- or NMS-sheep anti-mouse M280 Dynabeads overnight at 4 °C. The next day, the Dynabeads were washed three times and the pellets were dissolved in 50 µl of 2× sample buffer. Proteins were analyzed by western blotting as previously described.

Cell fractionation was performed by lysing macrophages that were seeded in 6-well plates (800,000 cells/well) in 200 µl of ice cold hypotonic lysis buffer (5 mM Tris–HCl, pH 7.4, 5 mM NaCl, 2 mM EDTA, 1 mM CaCl_2_, 1 mM MgCl_2_, 2 mM DTT + Proteinase Inhibitor Cocktail, Roche, 11697498001), after which they were spun down for 1 h at max speed (13200 rpm) at 4 °C. The pellet or membrane fraction was dissolved in 50 µl of 2× sample buffer. The cytosol fraction was dissolved 1:4 with 4× sample buffer, and twice the amount was loaded compared with that of the membrane fraction. Proteins were analyzed by western blotting as previously described.

### HEK-Blue cell experiments

HEK-Blue™ mTLR4 reporting cells were purchased through InvivoGen, were obtained by co-transfection of the murine TLR4, MD-2 and CD14 co-receptor genes and an inducible SEAP reporter gene into HEK293 cells (HEK-Blue™ mTLR4, hkb-mtlr4, InvivoGen), and were cultured according to the manufacturer’s protocol. As the SEAP reporter gene was placed under the control of an IFN-β minimal promoter fused to five NFκB and AP1-binding sites, stimulation with a TLR4 ligand activates NFκB and AP1, which induces the production of SEAP. SEAP production was measured using QUANTI-Blue (QUANTI-Blue™, rep-qb1, InvivoGen) according to the manufacturer’s protocol. Cells were stimulated with either 2 or 10 ng/ml LPS (Sigma), and all other TLR ligands (Mouse TLR1-9 Agonist Kit, tlrl-kit1mw, InvivoGen; TLR-specific ligands: Pam3CSK4 for TLR1/2, Poly(I:C) (HMW) for TLR3, LPS-EK for TLR4, FLA-ST for TLR5, FLS-1 for TLR6/2, and ODN1826 for TLR9) were stimulated at the concentrations described in the manufacturer’s protocols. Before stimulation, the cells were transfected with either Silencer^®^ Negative Control siRNA (Life Technologies, AM4611) or Silencer^®^ OGN siRNA (Life Technologies, L-090181-01) using Lipopfectamine^®^ 2000 (Invitrogen, 11668-030) according to manufacturer’s protocol. Forty-eight hours after transfection,0 the cells were seeded in 96-well plates and the TLR ligands were added.

### Bone marrow-derived macrophage isolation and experiments

Both tibias and femurs were collected from OGN WT and KO mice in ice cold PBS and stripped of muscles. After placing the stripped bones in 70% ethanol for approximately 45 s, they were again washed with PBS, after which the ends were removed and the inner bone marrow was flushed with a 25-G syringe filled with cold PBS. After flushing all of the bones, a single-cell suspension was obtained by pushing the suspension through a 100-µm Nylon cell strainer. Cells were spun down at 1200 rpm, placed in bacterial plates in RPMI 1640 with 15% LCM for cell culture, and differentiated for approximately 8–10 days; medium was added or replaced every 2–3 days. Experiments were performed after differentiation for 8–10 days. Cells were counted using a Burker Turk cell counting chamber, and 400,000 cells were seeded in 12-well plates. The cells were stimulated the next day, after the cells had adhered to the plastic, with 10 ng/ml LPS (Sigma). At the end of the experiments, the cells were directly harvested in RLT buffer for RNA isolation and sample buffer for immunoblotting.

### Human buffy coat preparation and Dynabead cell isolation

Blood samples were collected using 5-ml BD Vacutainer K2E (EDTA) tubes with erythrocyte lysis buffer (8.4 g NH_4_Cl + 0.84 g NaHCO_3_ in 1 L Aqua Dest, pH 7.2–7.4). Blood was spun down at 200 g with the brake off for 20 min. Platelet-rich plasma was removed, after which the cells were incubated for 3 min with 10 ml of ice cold erythrocyte lysis buffer. Next, the cells were spun down at 1500 rpm for 10 min. Erythrocyte lysis was repeated if erythrocytes were still present, because this would interfere with Dynabead cell isolation. One hundred microliters of Dynabeads^®^ M-280 Sheep Anti-Rabbit IgG (Invitrogen, 11203D) was incubated with 8 µg of anti-OGN antibody (Sigma HPA013132) overnight at 4ºC according to the manufacturer’s protocol in washing buffer [Ca^2+^- and Mg^2+^-free phosphate buffered saline (PBS) supplemented with 0.1% bovine serum albumin (BSA) and 2 mM EDTA at pH 7.4]. The next day, the beads were washed three times with washing buffer, after which they were incubated with the buffy coat lysates. The buffy coat lysates were washed twice with PBS with 0.1% BSA by centrifugation at 225*g* for 8 min at 2–8 °C and re-suspended at 1 × 10^8^ cells/ml in PBS with 0.1% BSA. One milliliter of cell suspension was incubated with 100 µl of beads at 2–8 °C for 30 min with gentle tilting and rotation. After washing the bead-bound cells twice, they were re-suspended in 100 μL of buffer for FACS analysis. The term input is referring to the complete human buffy coat samples that were used. Addition of the OGN-bound beads removed all OGN-positive leukocytes from these total buffy coats. The remaining fraction was referred to as OGN-negative. All fractions were analyzed by FACS. In addition, the phosphorylation of c-jun was determined by dissolving all fractions in sample buffer, after determining total protein content using Micro BCA™ Protein Assay Kit (Life Technologies, 23235)

### Human samples

For Immunohistochemical analysis, histological section was obtained from 16 patients suggestive of viral myocarditis and of 14 post-mortem ‘healthy controls’ manually selected based on clinicopathological reports from the host university hospital (Charité, Berlin, Germany) by an experienced pathologist (E. V.), who was not involved in the preclinical data acquisition. The ethical commission of Charité Germany approved the study. For western blot analysis, human endomyocardial biopsy samples suggestive of myocardial infarction of post-mortem ‘healthy controls’ were manually selected based on clinicopathological reports from the host university hospital (MUMC, Maastricht, The Netherlands) by an experienced pathologist (E. V.), who was not involved in the preclinical data acquisition. The ethical commission of MUMC Maastricht approved the study.

Buffy coats for western blot analysis and flow cytometry were collected from healthy volunteers after providing proper informed consent.

### Flow cytometry

Cells were stained with anti-CD3-FITC, CD66b-FITC, CD19-FITC, CD19-BV421, CD56-PE, and HLA-DR-V55 (BD Biosciences), and measured with FACS-Canto II (BD Biosciences). Results were analyzed with the FACSdiva software (BD Biosciences).

### RT-PCR

Real-time reverse transcriptase-polymerase chain reaction (RT-PCR) analysis was performed (Bio-Rad, Maastricht, The Netherlands) to determine the transcript levels of the following genes:

CVB3: forward primer ACGAATCCCAGTGTGTTTTGG
  reverse primer TGCTCAAAAACGGTATGGACAT at 63.9 °C;
IL-6: forward primer CAAAGCCAGAGTCCTTCAGAG
  reverse primer GCCACTCCTTCTGTGACTCC at 63.9 °C;
TNFα: forward primer CCACCACGCTCTTCTGTCTA
  reverse primer AGGGTCTGGGCCATAGAACT at 63.9 °C;
IL-1β: forward primer GTAATGAAAGACGGCACACC
  reverse primer TACCAGTTGGGGAACTCTGC at 63.9 °C;
IL-12: forward primer CTAGACAAGGGCATGCTGGT
  reverse primer TCTCCCACAGGAGGTTTCTG at 63.9 °C;
OGN: forward primer CCTGGAATCTGTGCCTCCTA
  reverse primer TCCAGGCGAATCTCTTCAAT at 63.9 °C;
XYLT2: forward primer AGAGTCTGGAGGTTGGTACTGAG
  reverse primer GCTACGGGCTCATCCAGTG at 61 °C;
GlcNact: forward primer GCTACTTCTAGAACCATTCTTGTCA
  reverse primer GCATAAGTTTCGTTGGTTCTGT at 61 °C;
Gsgalnact: forward primer GACTCGCCGAGGCTTTACTC
  reverse primer AGTCATAGCCCCAAAGTGGC at 61 °C;
Gluc-C5-Epimerase: forward primer GTGGAGTTGAAGGTGTGCCA
  reverse primer GTGAGGGGGTTTCTCGGTTA at 61 °C;
GAPDH: forward primer GGTGGACCTCATGGCCTACA
  reverse primer CTCTCTTGCTCAGTGTCCTTGCT at 63.9 °C.

The primer sequences of these genes were determined by the NCBI software analysis of Primer BLAST. The details of the sequences and thermal cycling conditions were according to the standard protocol. Data were acquired and analyzed with the IQ5 software (Bio-Rad, Maastricht, The Netherlands).

### Statistical analysis

The results represent the mean ± SEM unless otherwise indicated. For murine studies, D’Agistino and Pearson’s omnibus normality test was performed. Statistical significance was determined by unpaired Student’s *t* test and one-way ANOVA when the data were normally distributed. Wilcoxon, Mann–Whitney, and Kruskal–Wallis tests with Dunn’s multiple comparison tests were used for non-parametric data as indicated. The Gehan–Breslow–Wilcoxon test was used for survival analysis. Statistical analyses were performed with the GraphPad Prism software v5.0 with *p < 0.05, **p < 0.01, and ***p < 0.001.

## Electronic supplementary material

Below is the link to the electronic supplementary material.

Supplementary material 1 (PDF 590 kb)
