# Supplementary material for: A novel 72-kDa leukocyte-derived osteoglycin enhances the activation of toll-like receptor 4 and exacerbates cardiac inflammation during viral myocarditis
Source: Cell Mol Life Sci. 2016 Nov 23;74(8):1511–25. doi: 10.1007/s00018-016-2423-7 (PMC5357299; doi:10.1007/s00018-016-2423-7)
Supplement: Supplementary file 1 — Supplementary material 1 (PDF 590 kb) [file 18_2016_2423_MOESM1_ESM.pdf]

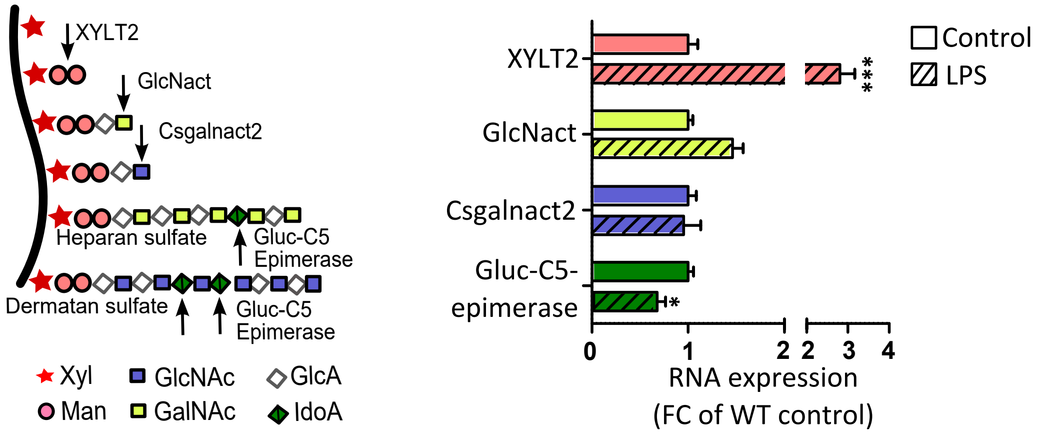

SUPPLEMENTARY FIGURE 1:
Bone marrow-derived macrophages (BMDMs) from WT mice were isolated and stimulated with LPS (10 ng/ml). The expression of glycosylation enzyme XYLT2 increased in response to LPS while gluc-C5-epimerase expression decreased (*$p < 0.05$; ***$p < 0.001$) *XYLT2: Xylosyl transferase; gluc-C5-epimerase: 2 D-glucuronyl C5-epimerase; Glcnact: glycosyltransferase; Csgalnact2: N-acetylgalactosaminyltransferases; Xyl: Xylose; Man: Mannose; GalNAc: N-acetylgalactosamine; GlcNAc: N-acetylglucosamine; GlcA: Glucuronic acid; IdoA: Iduronic acid*

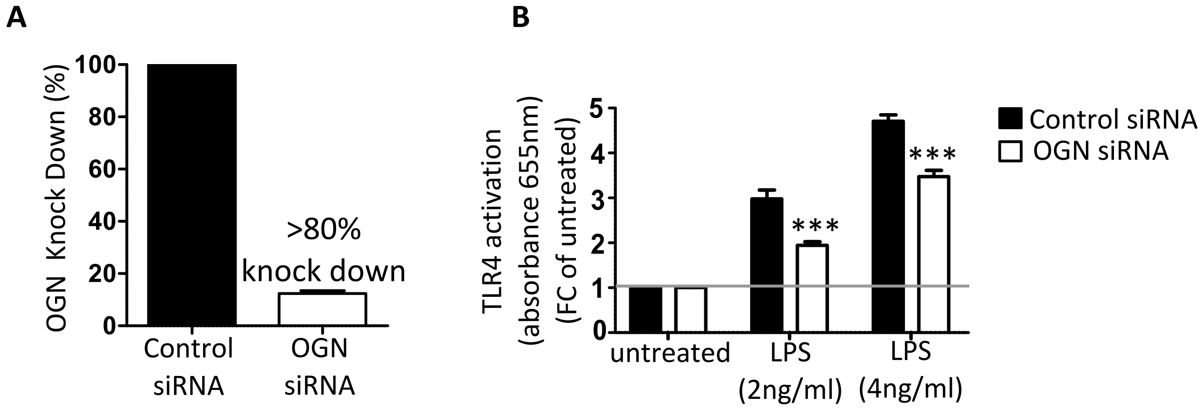

SUPPLEMENTARY FIGURE 2:
(A) HEK-Blue™ mTLR cells were treated with control siRNA and siRNA for OGN. RNA expression revealed a knockdown of approximately 80%. (B) HEK-Blue™ mTLR cells were treated with different doses of LPS. Knock down of OGN resulted in reduced TLR4 activation in all doses (***$p < 0.001$).

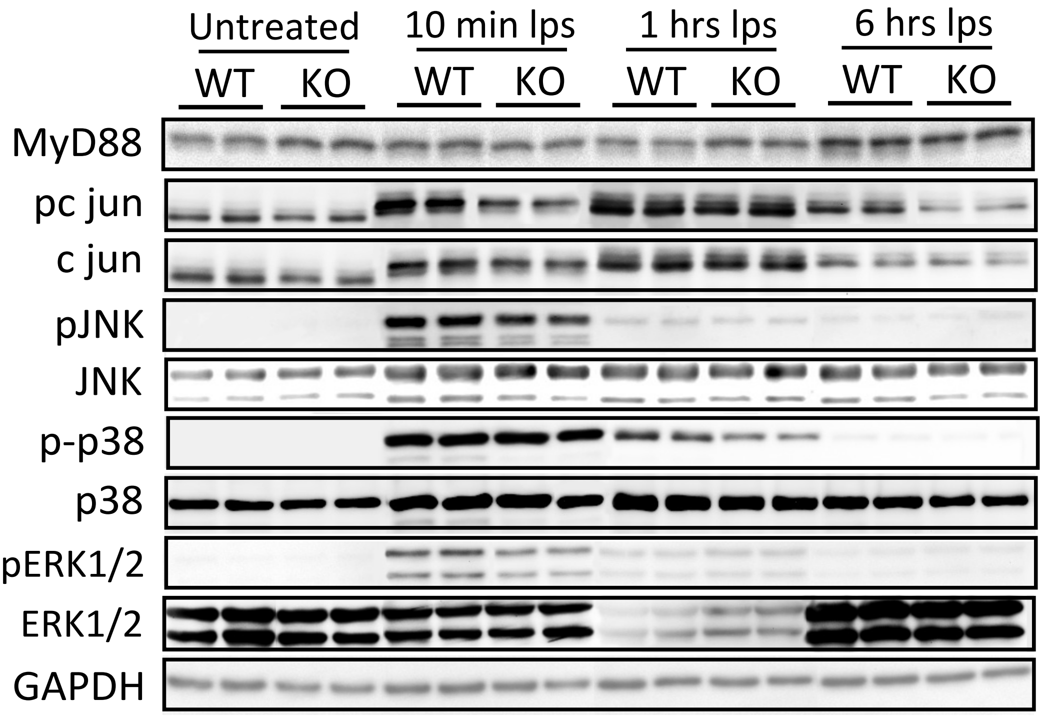

Supplementary Figure 3: OGN promotes TLR4 activation by enhancing MAPK-induced cytokine production, 1 hour time point included.
